# Improvement of androgenic alopecia by extracellular vesicles secreted from hyaluronic acid-stimulated induced mesenchymal stem cells

**DOI:** 10.1186/s13287-024-03906-x

**Published:** 2024-09-11

**Authors:** Hyun Geun Oh, Minyoung Jung, Seon-Yeong Jeong, Jimin Kim, Sang‑Deok Han, Hongduk Kim, Seulki Lee, Yejin Lee, Haedeun You, Somi Park, Eun A. Kim, Tae Min Kim, Soo Kim

**Affiliations:** 1 R&D Center, Brexogen Inc., 3F, 9, Beobwon-ro 8-gil, Songpa-gu, Seoul, 05855 Republic of Korea; 2 Institute of Green Bio Science and Technology, Seoul National University, 1447 Pyeongchang Daero, Pyeongchang, Gangwon-do 25354 Republic of Korea; 3 Graduate School of International Agricultural Technology, Seoul National University, Pyeongchang, Gangwon-do 25354 Republic of Korea

**Keywords:** Androgenetic alopecia, Wnt/β-Catenin signaling, Androgen receptor, Extracellular vesicles, Induced mesenchymal stem cells

## Abstract

**Background:**

Androgenetic alopecia (AGA) is a common form of hair loss. Androgens, such as testosterone and dihydrotestosterone, are the main causes of AGA. Extracellular vesicles (EVs) derived from mesenchymal stem cells (MSCs) can reduce AGA. However, preparing therapeutic doses of MSCs for clinical use is challenging. Induced pluripotent stem cell-derived MSCs (iMSCs) are homogenous and easily expandable, enabling scalable production of EVs. Hyaluronic acid (HA) can exert various functions including free radical scavenging, immune regulation, and cell migration. Herein, we examined whether hyaluronic acid (HA) stimulation of iMSCs could produce EVs with enhanced therapeutic outcomes for AGA.

**Methods:**

EVs were collected from iMSCs primed with HA (HA–iMSC–EVs) or without HA (iMSC–EVs). The characteristics of EVs were examined using dynamic light scattering, cryo-transmission electron microscopy, immunoblotting, flow cytometry, and proteomic analysis. In vitro, we compared the potential of EVs in stimulating the survival of hair follicle dermal papilla cells undergoing testosterone-mediated AGA. Additionally, the expression of androgen receptor (AR) and relevant growth factors as well as key proteins of Wnt/β-catenin signaling pathway (β-catenin and phosphorylated GSK3β) was analyzed. Subsequently, AGA was induced in male C57/BL6 mice by testosterone administration, followed by repeated injections of iMSC–EVs, HA–iMSC–EVs, finasteride, or vehicle. Several parameters including hair growth, anagen phase ratio, reactivation of Wnt/β-catenin pathway, and AR expression was examined using qPCR, immunoblotting, and immunofluorescence analysis.

**Results:**

Both types of EVs showed typical characteristics for EVs, such as size distribution, markers, and surface protein expression. In hair follicle dermal papilla cells, the mRNA levels of *AR, TGF-β*, and *IL-6* increased by testosterone was blocked by HA–iMSC–EVs, which also contributed to the augmented expression of trophic genes related to hair regrowth. However, no notable changes were observed in the iMSC–EVs. Re-activation of Wnt/β-catenin was observed in HA–iMSC–EVs but not in iMSC–EVs, as shown by β-catenin stabilization and an increase in phosphorylated GSK3β. Restoration of hair growth was more significant in HA–iMSC–EVs than in iMSC–EVs, and was comparable to that in mice treated with finasteride. Consistently, the decreased anagen ratio induced by testosterone was reversed by HA–iMSC–EVs, but not by iMSC–EVs. An increased expression of hair follicular β-catenin protein, as well as the reduction of AR was observed in the skin tissue of AGA mice receiving HA–iMSC–EVs, but not in those treated with iMSC–EVs.

**Conclusions:**

Our results suggest that HA–iMSC–EVs have potential to improve AGA by regulating growth factors/cytokines and stimulating AR-related Wnt/β-catenin signaling.

**Supplementary Information:**

The online version contains supplementary material available at 10.1186/s13287-024-03906-x.

## Background

Androgenetic alopecia (AGA) is a common form of hair loss which is affected by genetic, hormonal, and environmental factors, as well as aging [1, 2]. Notably, the hair follicles of patients with AGA has a higher sensitivity to androgens such as dihydrotestosterone (DHT). In hair follicles, testosterone is converted to DHT by 5α-reductase and prolonged exposure to DHT makes hair follicles shrink, eventually leading to hair loss [3]. Several strategies for AGA are available, but only finasteride and minoxidil are approved by the US Food and Drug Administration [4]. However, prolonged use of finasteride can cause sexual dysfunction, while minoxidil has side effects such as irritation, swelling, and lightheadedness [5–7].

The Wnt/β-catenin pathway plays an essential role in the hair follicle development from dermal papilla cells (DPCs) [8–10]. In the presence of Wnt ligands, the phosphorylation of β-catenin by glycogen synthase kinase 3β (GSK3β) is inhibited, stabilizing β-catenin. Without the Wnt ligand, GSK3β phosphorylates β-catenin leading to the degradation and ubiquitination of β-catenin [11]. In patients with AGA, DHT inhibits the Wnt/β-catenin pathway by activating GSK3β, contributing to the inhibition of hair follicular stem cell differentiation in DPCs [12]. Consistently, the inhibition of GSK3β via phosphorylation (at Ser9) by human placental extract caused β-catenin stabilization and promoted the hair-inductive capacity of DPCs [13]. Collectively, activation of the Wnt/β-catenin pathway by blocking GSK3β activity is required to block AGA progression.

Extracellular vesicles (EVs), which are nano-sized particles released from almost all cell types, play an essential role in cell-to-cell communication [14]. In addition, the content of EVs in body fluids can provide biological information about the original tissues, making EVs invaluable tools as non-invasive diagnostic markers [15]. Importantly, stem cell-derived EVs have potential for therapeutic purposes in various diseases due to their immune-modulatory, anti-inflammatory, anti-apoptotic, and pro-survival roles [14, 16, 17]. The effect of mesenchymal stem cell (MSC)-derived EVs (MSC-EVs) on hair growth has been previously reported [18, 19]. Rajendran et al. [18] showed that EVs derived from mouse bone marrow-derived MSCs promote hair regrowth. Additionally, exosomes from human subcutaneous tissue supported the proliferation and migration of DPCs in vitro, and inhibited GSK3β activity via phosphorylation of GSK3β at Ser9, resulting in improved hair regrowth in DHT-induced AGA mice [19].

Despite the potential of MSCs for therapeutic purposes, they are not feasible for industrial or clinical applications, mostly due to limited proliferation in vitro, heterogenicity, and potential immune responses due to their allogenic origin [20]. To overcome these problems, induced pluripotent stem cell-derived MSCs (induced MSCs; iMSCs) can be used as an alternative to conventional MSC-based therapies, because iMSCs are homogenous and easily expandable [21–23]. In addition, the cellular characteristics of iMSCs are different from those of conventional MSCs and iMSCs may have a better potential for treating immune or inflammatory diseases [24, 25].

Hyaluronic acid (HA) is one of the most widely used biodegradable polymers because of its excellent hydration and lubrication properties. Importantly, HA has diverse physiological functions, including free radical scavenging, immune regulation, and cell migration [26–28]. HA has been used for skin tissue repair and wound healing [29, 30]. In this study, we hypothesized that HA stimulation of iMSCs could produce EVs with improved therapeutic outcomes in AGA mice. We report that HA–iMSC–EVs, but not iMSC-EV, reduced AGA by stabilizing β-catenin, inhibited protein expression of androgen receptor, as well as stimulating growth factors/cytokines responsible for hair follicle cycle.

## Materials and methods

### Isolation of EVs

iMSCs were generated from induced pluripotent stem cells (iPSC; STEMCELL Technologies Inc., Vancouver, Canada) as described in our previous study [31]. Briefly, iPSCs were maintained for 2 weeks in Dulbecco’s modified Eagle's medium (DMEM) high glucose supplemented with 15% FBS (ATCC, Manasas, VA, USA) and 1% antibiotic-antimycotics (Thermo Fisher Scientific, Waltham, MA, USA), and the medium was changed every other day. Cells were passaged to gelatin-coated tissue culture vessels (EMD Millipore, Billerica, MA, USA) using TryPLE Express. The established iMSCs were cultured in high-glucose DMEM (HyClone, Chicago, IL, USA) supplemented with 15% fetal bovine serum (FBS; HyClone) and 1% antibiotic–antimycotic solution (Thermo Fisher Scientific) at 37 °C in a humidified incubator containing 5% CO_2_. At 90% confluence, cells were detached using TrypLE Express (Thermo Fisher Scientific) and seeded at a density of 10,000 cells/cm^2^. The next day, the cells were stimulated with or without 40 μg/mL HA (Sigma-Aldrich, St Louis, MO, USA) for 24 h. After being washed with Dulbecco’s Phosphate Buffered Saline (HyClone), cells were cultured in phenol red-free DMEM (Gibco, Waltham, MA, USA) supplemented with 15% EV-depleted FBS. EV-depleted FBS was prepared as previously described [32]. After 3 d of incubation, the culture medium was harvested and HA–iMSC–EVs were isolated by ultracentrifugation, as previously described [33].

### Nanoparticle tracking analysis and cryo-transmission microscopy analyses of EVs

Nanoparticle tracking analysis (NTA) was carried out using Zetaview^®^BASIC NTA-Nanoparticle Tracking (Particle Metrix, Inning am Ammersee, Germany) to confirm size distribution and concentration of EVs. The values for the standard controls were set as follows; Sensitivity: 80, Frame Rate: 30, Shutter: 100, Temperature: 23 °C. Cryo-transmission electron microscopy (Cryo-TEM) was performed to confirm the morphology of EVs using a Talos L120C FEI transmission electron microscope (Thermo Fisher Scientific) as described in our previous study [32]. HA–iMSC–EV suspension (4 μL) was placed on a grid and blotted for 90 s at 100% humidity and 4 °C. HA–iMSC–EVs were visualized at 36,000× magnification at 120 kV.

### Bioinformatic analysis of HA–iMSC–EVs

EVs (50 μg) were lysed by RIPA buffer (Thermo Fisher Scientific) buffer and the lysates were transferred into 10 kDa filter and centrifuged at 12,000*g* for 5 min (three times). The proteins in the samples were quantified using a Qubit fluorometer (Thermo Fisher Scientific). Twenty micrograms of protein was digested in DTT and iodoacetamide and then dried using a SpeedVac concentrator (Hanil Science Medical, Daejeon, Korea). The protein samples were desalted using C18 spin columns (Thermo Fisher Scientific), washed with 0.1% formic acid in LC–MS grade water (Thermo Fisher Scientific) and eluted with 0.1% formic acid in 50% LC–MS grade acetonitrile (Thermo Fisher Scientific). LC–MS/MS was performed using a Vanquish Neo UHPLC System (Thermo Fisher Scientific) and tryptic peptides were identified and quantified using a Proteome Discoverer (Thermo Fisher Scientific). The UniProt database was used to identify proteins in the samples. For proteome analysis, protein set sorting of HA–iMSC–EVs and iMSC–EVs was performed with abundance ranks of less than 400 and adjusted *p* values of less than 0.05. The identification of proteins was performed with 1.0% of false discovery rate. Pathway analyses were performed using the Kyoto Encyclopedia of Genes and Genomes database (CELLKEY Inc., Seoul, Korea).

### Hair follicle dermal papilla cell (HFDPC) culture

The HFDPC were purchased from PromoCell (Heidelberg, Germany). HFDPC were cultured in follicle dermal papilla cell media (PromoCell) supplemented with 4% fetal calf serum, 0.4% bovine pituitary extract, 1 ng/mL basic fibroblast growth factor, 5 μg/mL insulin and 1% antibiotic–antimycotic solution. The HFDPC were cultured according to the manufacturer’s instruction. The cultured cells were maintained at 37 °C and 5% CO_2_ in a humidified incubator and used within 10 passages.

### Western blot analysis

HFDPC lysates were prepared using RIPA lysis buffer (Thermo Fisher Scientific) containing complete mini EDTA-free protease inhibitor tablets (Roche, Basel, Switzerland) for 30 min and centrifuged at 12,000 rpm for 10 min. For the fractionation of nuclear and cytoplasmic, NE-PER Nuclear and Cytoplasmic Extraction Reagents (Thermo Fisher Scientific) was used and the fractionation was performed according to the manufacturer’s protocol. Protein was quantified using a BCA acid assay according to the manufacturer's instructions (Thermo Fisher Scientific). Proteins were separated by 4–15% SDS-PAGE and transferred to PVDF membranes (Bio-Rad, Hercules, USA) which were used to detect all proteins. The membrane was blocked with Everyblot blocking buffer (Bio-Rad) and incubated overnight with the primary antibody at 4 °C, which was followed by incubating with secondary antibody against immunoglobulin (IgG) at 23–24 °C for 60 min. The primary antibodies against CD63 (Abcam, Cambridge, UK), CD81 (Invitrogen, Waltham, Massachusetts, USA), Tumor Susceptibility Gene 101 (TSG101, Invitrogen), glyceraldehyde 3-phosphate dehydrogenase (GAPDH; Abcam), AR (Santa Cruz Biotechnology, Dallas, USA), β-catenin (Cell Signaling Technology, Danvers, USA), GSK3β (Cell Signaling Technology), phospho-GSK3β (Cell Signaling Technology), Cyclin D1 (Cell Signaling Technology) and Lamin A/C (Abcam) were used. Information about the antibodies used in western blots are listed in Additional file 1: Table S1. Secondary antibody-conjugated peroxidase (Abcam) activity was visualized by enhanced chemiluminescence. ImageJ 1.54 h software using a ChemiDoc system (Bio-Rad) was used to quantify the immunoblots.

### Flow cytometry

iMSC–EVs and HA–iMSC–EVs were stained using the Human MACSPlex Exosome Kit (Miltenyi Biotec, Bergisch Gladbach, Germany) according to the manufacturer’s protocol and analyzed using an Attune NxT flow cytometer (Thermo Fisher Scientific).

### cDNA synthesis and real-time quantitative polymerase chain reaction (qPCR)

Total RNA was extracted from HFDPC using the TRIzol reagent (Invitrogen). cDNA was synthesized from 1 μg of purified total RNA using a AccuPower^®^ CycleScript RT PreMix (dT20) (Bioneer, Daejeon, Korea) according to the manufacturer’s instructions. The primers used for qPCR analysis are listed in Additional file 1: Table S2. qPCR was performed using Power SYBR green PCR master mix (Thermo Fisher Scientific) containing 0.5 μM forward and reverse primers, according to the manufacturer’s manual. Real-time PCR was performed on a Real-time PCR Detection System (Bio-Rad). The experiment was repeated three times. Data were analyzed using the 2^−ΔΔCT^ method, and the expression of each gene was normalized to that of *GAPDH*.

### Animals and in vivo experiments

Animal care and procedures were approved by the Institutional Animal Care and Use Committee of Seoul National University (#SNU-230116-4-1). Male six-week-old C57BL/6N mice weighing 18–19 g were purchased from Koatech Inc. and housed at 23–24 °C with a 12/12 h light/dark cycle for two weeks. Twenty-five Mice were randomly divided into five groups (five mice per group), including group 1 (vehicle control group) which received a 50% EtOH for topical application and DPBS for subcutaneously injection; group 2 (testosterone-treated group) received 0.5% testosterone propionate topically application for 27 d (Tokyo Chemical Industry, Tokyo, Japan) that was prepared in 50% ethanol; group 3 (positive control) received 0.5% testosterone topical application and was then subcutaneously injected with 1 mg/kg finasteride; and groups 4 and 5 (HA–iMSC–EVs and iMSC–EVs) were treated with 0.5% testosterone, followed by subcutaneous injection with 0.2 mg/kg of HA–iMSC–EVs and iMSC–EVs, respectively. To minimize the variance that can arise from the order of treatments, administration was performed in following order: (1) finasteride (2) iMSC–EVs, (3) HA–iMSC–EVs, and 4) D-PBS. Each injection procedure took one minute. The number of mice per group was determined by the information in previous study [34, 35]. Testosterone was topically applied daily, and finasteride, iMSC–EVs, and HA–iMSC–EVs were subcutaneously injected every other day for 27 d. One day before the experiment, back hair of mice was clipped from the dorsal surface of each mouse with an electric shaver and hair removal cream (Hyundai Pharm, Seoul, Korea) under anesthesia using 2% isoflurane for 5 min. Hair growth was photographed on days 1, 13, 20, and 27. During this study, animals were monitored daily for the criteria for human endpoints: (1) when movement is significantly reduced, (2) situations in which food or water intake is difficult, (3) when body weight has decreased by more than 20%, (4) when the response is significantly reduced, or (5) if the hair becomes markedly rougher. None of the animals were found adversely affected. Data from all animals was included. Oh HK was aware of the group allocation. All procedures for animal experiments were in accordance with the ARRIVE guidelines for the reporting of animal experiments.

### Immunohistochemistry

The mice were euthanized by CO_2_ inhalation and the dorsal skin was sectioned. Skin tissue was fixed in 4% paraformaldehyde and embedded in paraffin to obtain longitudinal sections. The paraffin blocks were cut into 5 μm thick sections. For hematoxylin and eosin (H&E) staining, sections were deparaffinized in xylene for 40 min, rehydrated in serially graded ethanol (100%, 95%, and deionized H_2_O) and then stained with hematoxylin for 13 min, followed by washes for 10 min, and eosin staining for 2 min. They were dehydrated three times for 2 min in serially graded ethanol (95% and 100%). Slides were mounted with xylene. For immunofluorescence histochemistry, the primary antibodies used were rabbit anti-β-catenin (Cell Signaling Technology) and mouse anti-AR (Santa Cruz Biotechnology). The secondary antibodies used were goat anti-rabbit IgG conjugated with Alexa Fluor 488 (Abcam) and goat anti-mouse IgG conjugated with cyanine5 (Invitrogen). Information about the antibodies used in immunohistochemistry were listed in Additional file 1: Table S3. Immunofluorescence images were obtained using a Nikon Eclipse T*i*2-U fluorescence microscope and analyzed using Nikon imaging software-elements program 5.2 (Nikon, Japan). Immunofluorescence-stained hair follicles were analyzed using individual hair follicles randomly selected from five mice per group (n = 21–26 hair follicles from five mice).

### Statistical analyses

Statistical analyses were performed using Prism 10 software (GraphPad Prism, Boston, MA, USA). Data are presented as means ± standard error of the mean. One-way analysis of variance, followed by post hoc Tukey’s test, was used. Differences with a *P* < 0.05 were considered statistically significant.

## Results

### Characterization of HA–iMSC–EVs

**Fig. 1:**
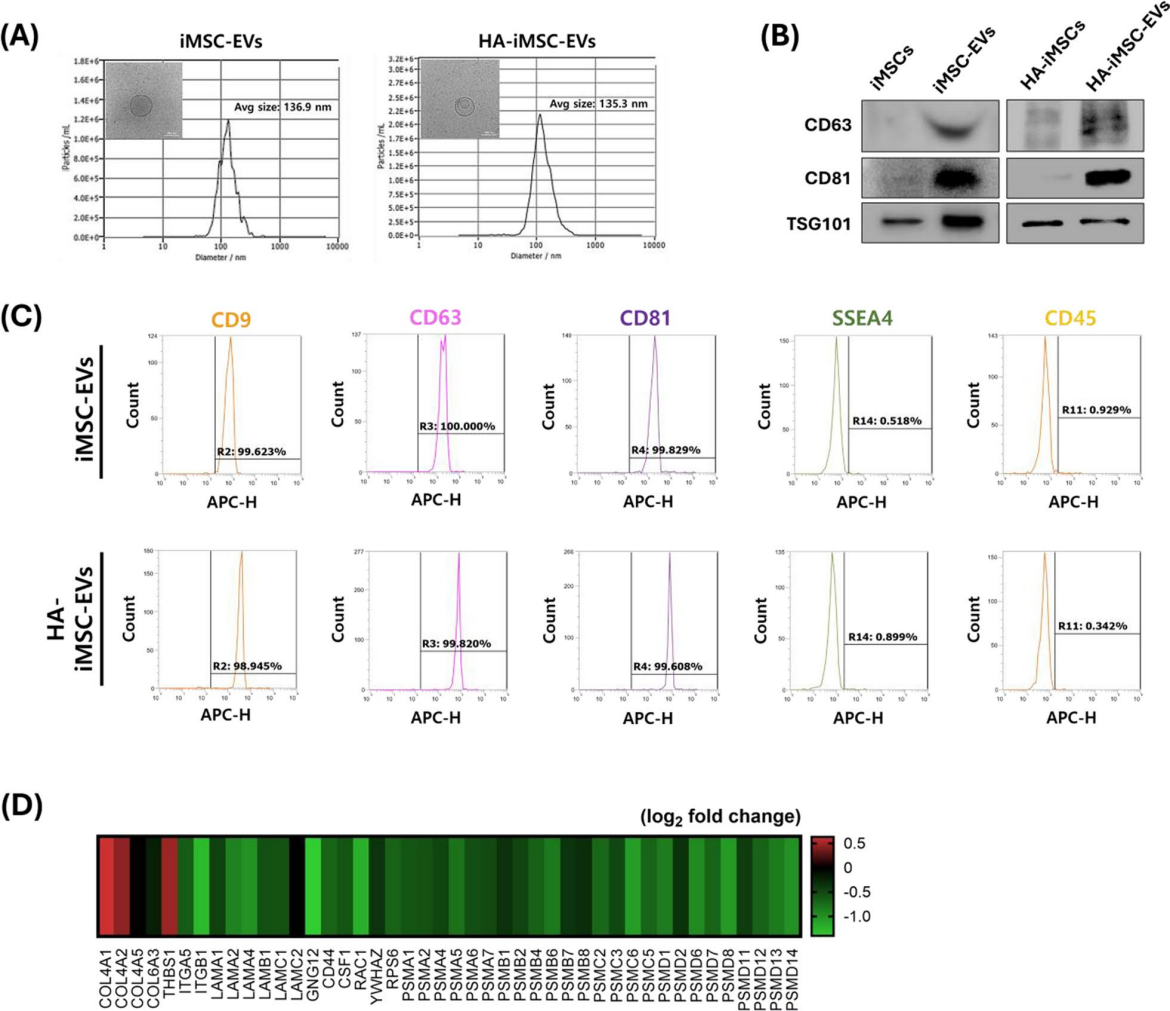
Characterization of iMSC–EVs and HA–iMSC–EVs. **A** HA–iMSC–EVs were measured the average size is 135.3 nm via NTA. (inset) Representative image of HA–iMSC–EVs observed using Cryo-TEM. Scale bar = 100 nm. **B** Immunoblotting analysis of HA-iMSCs and HA–iMSC–EVs for markers of extracellular vesicles. Uncropped western blot images are shown in Additional file 1: Fig. S1. **C** Flow cytometric analysis of iMSC–EVs and HA–iMSC–EVs. **D** Representative heatmap for analysis of differentially expressed proteins of HA–iMSC–EVs by LC–MS/MS analysis. The differentially expressed proteins in HA–iMSC–EVs against those from iMSC–EVs were marked in red and green, respectively. Enriched pathways, p-values, and fold change are shown in Additional file 1: Table S4. Cryo-TEM, Cryo-transmission electron microscopy; EV, extracellular vesicle; HA, hyaluronic acid; iMSC, induced pluripotent stem cell-derived mesenchymal stem cell; NTA, nanoparticle tracking analysis

To confirm the specifications of the iMSC–EVs and HA–iMSC–EVs, we measured the size of the EVs using NTA and observed their morphology using Cryo-TEM. The average diameters of iMSC–EVs and HA–iMSC–EVs were 136.9 and 135.3 nm, respectively (Fig. 1A). The isolated EVs were spherical in shape with a clear membrane, as determined using Cryo-TEM. (Fig. 1A, inset). Both the EV types expressed CD63, CD81, and TSG101 (Fig. 1B). Flow cytometry revealed that iMSC–EVs and HA–iMSC–EVs tested positive for CD9, CD63, and CD81, which are typical extracellular vesicle surface markers, but negative for stage-specific embryonic antigen-4 (SSEA4) and CD45 (Fig. 1C). These results indicate that HA–iMSC–EVs met the general characteristics of EVs derived from cultured cells [31].

Next, we analyzed the proteomic profiles and potential pathways associated with HA–iMSC–EVs. As shown in Fig. 1D, 44 differentially expressed genes were associated with extracellular matrix-receptor interaction (ECM), PI3kinase/AKT signaling, and the proteasome (Additional file 1: Table S4).

### HA–iMSC–EVs modulated androgenic alopecia related-mRNA and protein expression in HFDPCs

To establish the appropriate dose of EVs, we compared the effects of iMSC–EVs and HA–iMSC–EVs on the survival of HFDPCs undergoing testosterone-induced AGA in vitro. The viability of HFDPC was reduced by testosterone, which was reversed by HA–iMSC–EVs (under 25 and 50 μg/mL, Fig. S2). In contrast, no increase in the number of iMSC–EVs was observed. Among three concentrations of HA–iMSC–EVs (10, 25, and 50 μg/mL), we determined to use 25 μg/mL for following cell experiments because this concentration was the minimum level that showed statistical significance (*p* < 0.05).

**Fig. 2:**
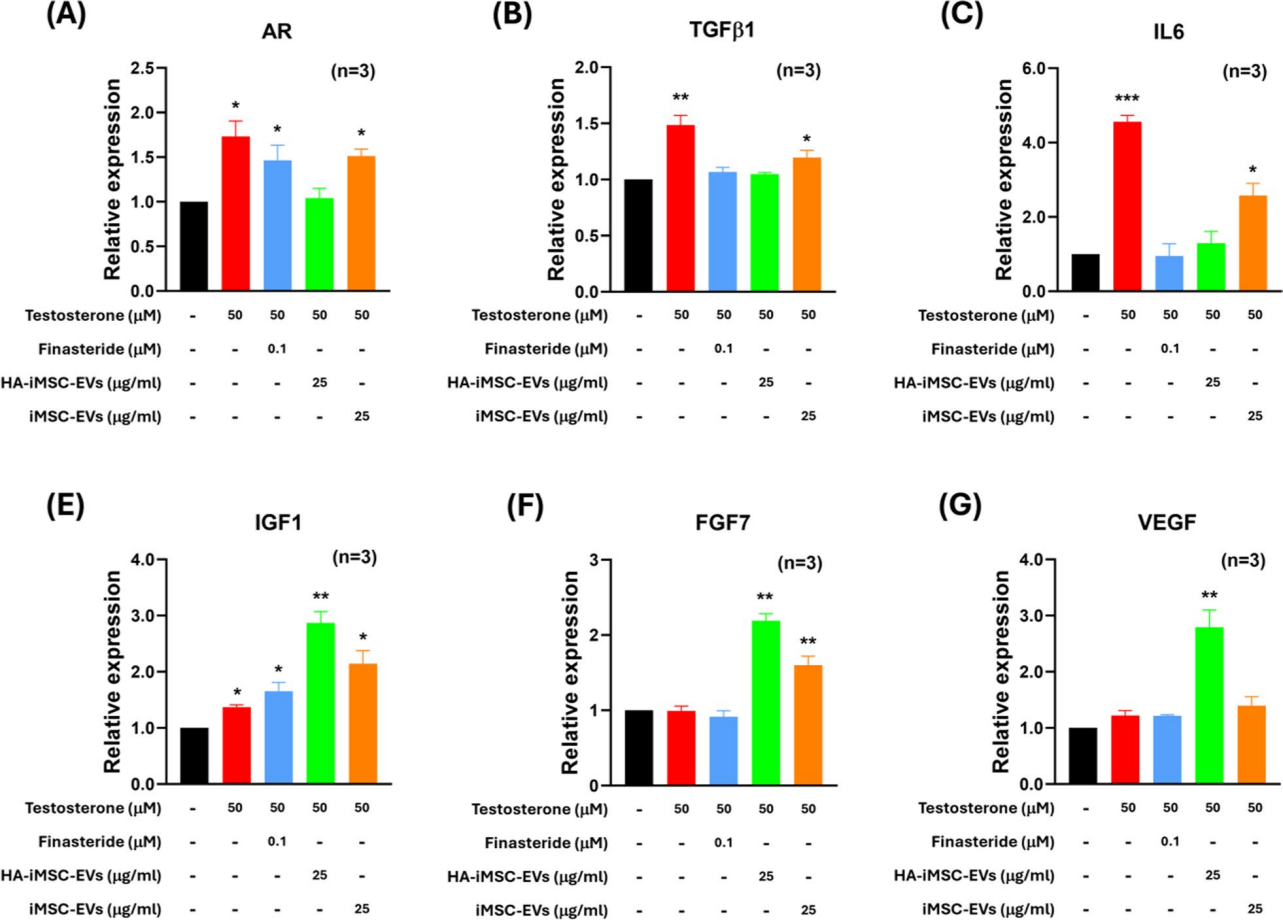
HA–iMSC–EVs modulated androgenic alopecia related-mRNA expression in HFDPC. **A**–**E** The mRNA expression was measured after being treated with 50 μM testosterone and each testing articles including finasteride (100 nM), HA–iMSC–EVs or iMSC–EVs (25 μg/mL each) for 24 h. Cells not treated with testosterone was used as the normal control. Results were from three replicates. *p < 0.05, **p < 0.01 versus control. EV, extracellular vesicle; HA, hyaluronic acid; HFDPC, hair follicle dermal papilla cell; iMSC, induced pluripotent stem cell-derived mesenchymal stem cell

A variety of growth factor-related and Wnt/β-catenin signaling pathways have been implicated in the hair cycling process and in hair follicle regeneration [36, 37]. Thus, we analyzed the mRNA expression of genes that are responsible for hair loss in HDFPC; *AR, TGFβ1, IL-6, IGF1, FGF7,* and *VEGF*. As shown in Fig. 2, the expression of *AR, TGFβ1,* and *IL-6*, which cause hair loss and converting hair follicles to catagen, were increased by testosterone (Fig. 2A–C). However, this increase was reversed by the HA–iMSC–EVs. The number of cells treated with finasteride or iMSC–EVs only partially decreased. IGF1, FGF7, and VEGF are critical for the maintenance of the anagen phase and stimulation of hair growth. The effect of testosterone on the expression of these growth factors was minimal. In contrast, HA–iMSC–EVs increased the mRNA expression of these growth factors more than two-fold (Fig. 2D–F). Finasteride and iMSC–EVs also partially increased IGF1 and FGF7, respectively.

**Fig. 3:**
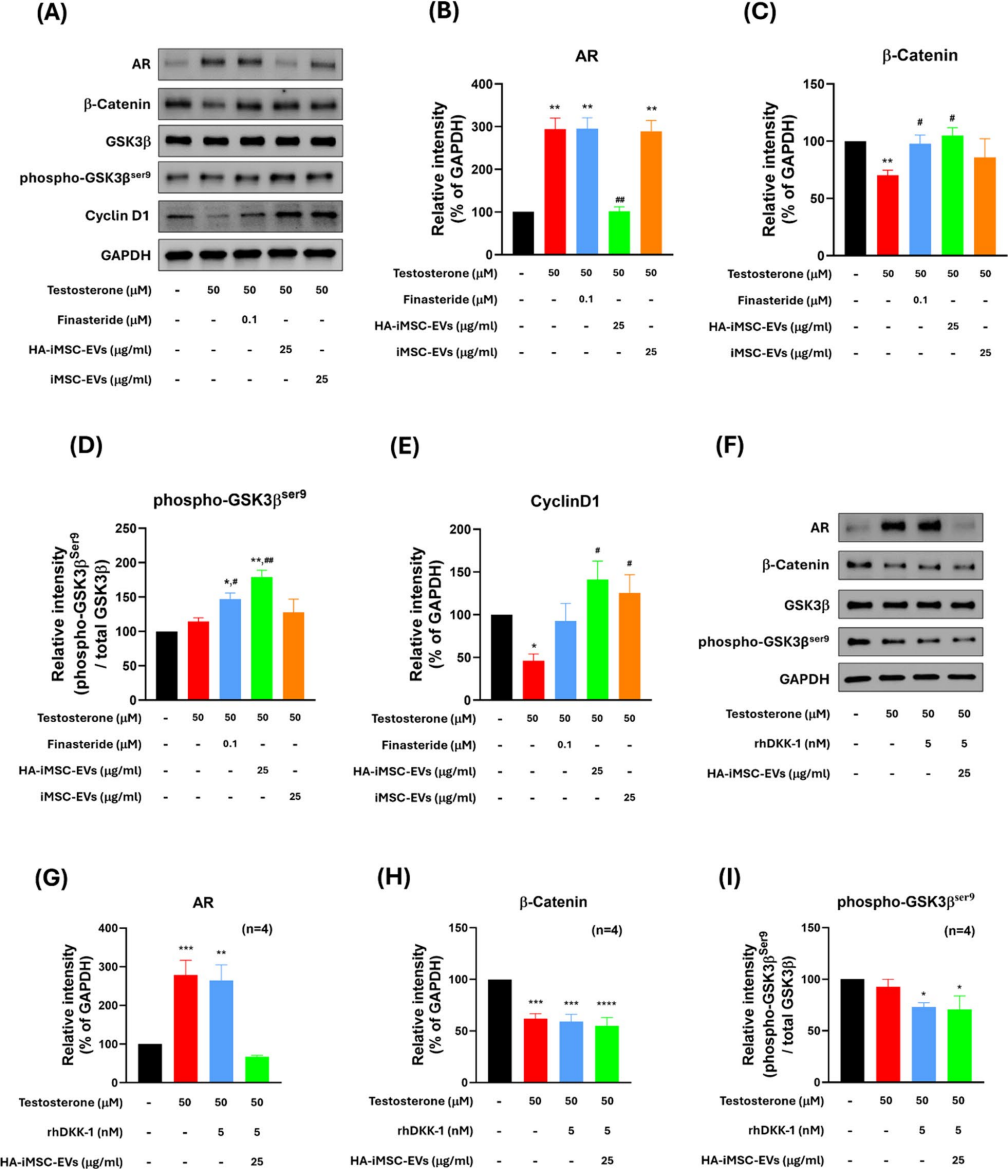
HA–iMSC–EVs regulated AR-related Wnt/β-catenin signaling and proliferation in HFDPC. **A**–**E** The protein expression was detected with 50 μM testosterone in the presence of 100 nM finasteride, 25 μg/mL HA–iMSC–EVs or 25 μg/mL iMSC–EVs for 24 h in HFDPC. **A** A typical western blotting result showing that HA–iMSC–EVs reduced AR expression and alleviated testosterone-induced alopecia. **B**–**E** The expression of each protein was normalized against that of GAPDH (N = 4). Uncropped western blot images are shown in Additional file 1: Fig. S3. **F**–**I** Inhibition of anti-AGA effects of HA–iMSC–EVs by rhDKK-1. HDFPCs were treated with 50 μM testosterone in the presence of 5 nM rhDKK-1 or 25 μg/mL HA–iMSC–EVs for 24 h. The expression of AR, β-catenin, and phospho-GSK3β^ser9^ was normalized against that of GAPDH (N = 4). Uncropped western blot images are shown in Additional file 1: Fig. S4. The protein bands were measured by the ImageJ software and plotted as mean ± SEM. *p < 0.05, **p < 0.01 versus control group. ^#^*p* < 0.05, ^##^*p* < 0.01 versus testosterone-only group. EV, extracellular vesicle; HA, hyaluronic acid; HFDPC, hair follicle dermal papilla cell; iMSC, induced pluripotent stem cell-derived mesenchymal stem cell

Next, western blotting was performed to observe the change of Wnt/β-catenin signaling molecules as well as proliferation marker proteins in HFDPC. Consistent with the mRNA expression levels, the expression of AR protein was increased by testosterone, which was reversed by HA–iMSC–EVs. No reduction was observed in cells treated with finasteride or iMSC–EVs (Fig. 3B). The phosphorylation of GSK3β at Ser9 residue (phospho-GSK3β^Ser9^) was augmented by finasteride or HA–iMSC–EVs (Fig. 3D), while no change was observed in iMSC-EV-treated cells.

Consistently, the expression of β-catenin was reduced by testosterone, which was then restored by finasteride or HA–iMSC–EVs (Fig. 3C). Testosterone treatment decreased cyclin D1 expression, which was subsequently restored by HA–iMSC–EVs and iMSC–EVs (Fig. 3E). Figure 3A shows that the decrease of β-catenin from both cytoplasm and nucleus was recovered by either Finasteride or HA–iMSC–EVs. Additionally, the reduction of nuclear translocation of β-catenin by testosterone was reversed by finasteride or HA–iMSC–EVs (Fig. S5A, B). Collectively, HA–iMSC–EVs have potential to repress AR expression and support the growth of HFDPC by reactivating Wnt/β-catenin pathway.

To further verify the role of HA–iMSC–EVs in activating Wnt/β-catenin pathway, a loss of function study was conducted using recombinant human Dickkopf-1 (rhDKK-1), an Wnt antagonist. Indeed, DKK-1 is a pathological mediator that induces hair follicles into the catagen, causing male pattern hair loss [38]. Additionally, DKK-1 has been reported to downregulate phospho-GSK3β^Ser9^ and accelerate β-catenin degradation in several cell types, such as keratinocytes and hippocampal neurons [39, 40]. HA–iMSC–EVs suppressed AR expression, regardless of the presence of rhDKK-1 (Fig. 3B, G). In contrast to the results shown in Fig. 3C, D, however, HA–iMSC–EVs did not affect the expression of β-catenin and phospho-GSK3β^ser9^ upon co-treatment with DKK-1 (Fig. 3H–I). Therefore, AR-related AGA therapeutic effect by HA–iMSC–EVs is dependent of Wnt/β-catenin signaling.

### HA–iMSC–EVs restored hair regrowth and hair follicle cycles in testosterone-treated C57BL/6N mice in vivo

**Fig. 4:**
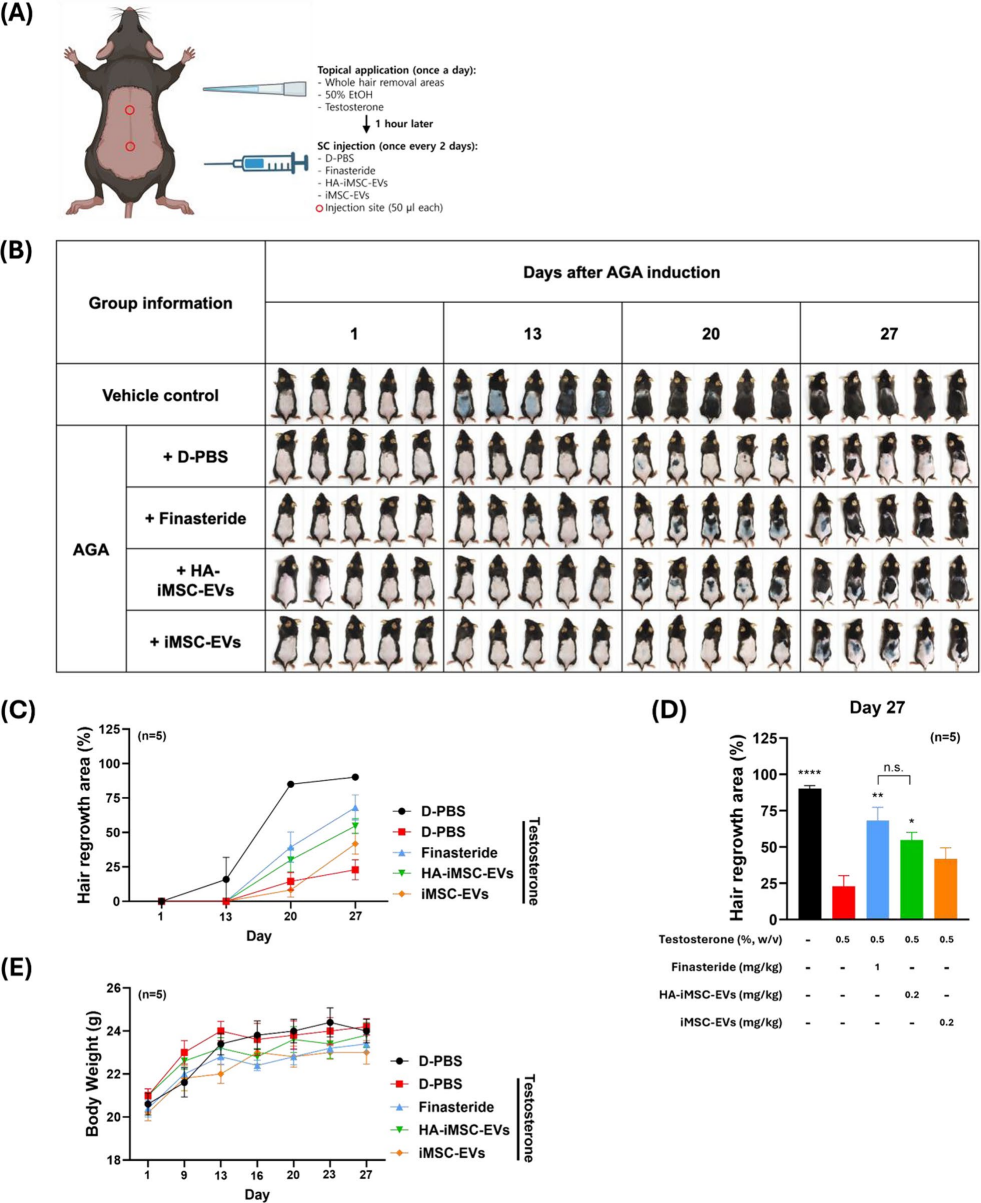
HA–iMSC–EVs restored hair regrowth and hair follicle cycles in testosterone-induced AGA mice. **A** Experimental design. **B** Images of the change of hair growth. Vehicle control denotes mice received topical application of 50% ethanol followed by repeated SC injection of D-PBS. AGA mice received topical application of testosterone followed by repeated SC injection of designated articles. **C**, **D** Quantification of hair regrowth area. ImageJ software was used and the relative area of hair growth against those at D1 was obtained. *p < 0.05, **p < 0.01, ****p < 0.0001 versus testosterone-only group **E** Body weight change. AGA, androgenetic alopecia; D-PBS, Dulbecco’s Phosphate Buffered Saline; EV, extracellular vesicle; HA, hyaluronic acid; iMSC, induced pluripotent stem cell-derived mesenchymal stem cell; SC, subcutaneous

Next, we compared the potential of HA–iMSC–EVs and iMSC–EVs for hair growth in testosterone-induced AGA mice. Testosterone (0.5%) was topically applied to the shaved skin of C57BL/6N mice once a day, and finasteride or EVs were injected subcutaneously every other day for 27 d (Fig. 4A). The dorsal hair was removed one day before drug treatment, and follow-up images were recorded at weekly intervals starting on day 13 (Fig. 4B) [41]. In normal mice (that received 50% ethanol), hair growth at day 13 was obvious, and became almost complete in all mice on day 20. In contrast, the hair growth was significantly reduced in testosterone-induced AGA mice, showing only 23.0% of hair grew area even on day 27 (Fig. 4B, D). On day 20, hair growth was promoted more in HA–iMSC–EVs than in iMSC–EVs (Fig. 4B). On day 27, the area of hair growth was higher in HA–iMSC–EVs than in iMSC–EVs and was comparable to those that received finasteride during the experimental period (Fig. 4B–D).

**Fig. 5:**
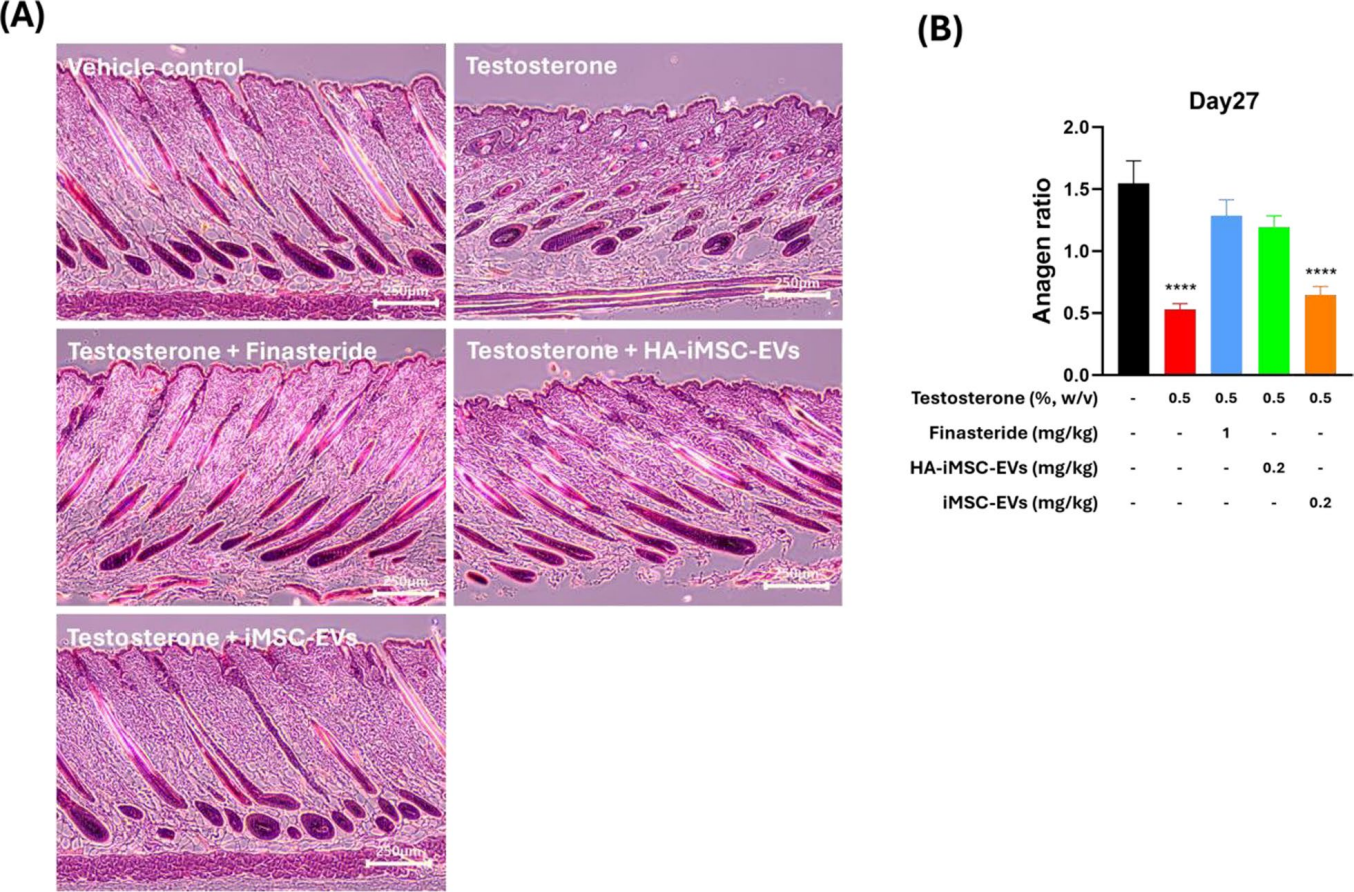
Histological investigation of the enhancement of telogen-to-anagen transition by HA–iMSC–EVs or iMSC–EVs. **A** Representative hematoxylin and eosin (H&E) stained images of the skin section on day 27. **B** Anagen:non-anagen (catagen/telogen) ratio in the skin sections. Data was collected from at least 92 hair follicles randomly selected from five mice per group. Progression of the hair follicle cycle on day 27 was quantitatively evaluated. ****p < 0.0001 versus vehicle control group. Scale bars = 250 μm. EV, extracellular vesicle; HA, hyaluronic acid; iMSC, induced pluripotent stem cell-derived mesenchymal stem cell

To investigate whether HA–iMSC–EVs can improve hair regrowth and induce the anagen phase in AGA mice, dorsal skin from each group were collected at 27 d and subjected to H&E staining. Histological analysis revealed that testosterone treatment reduced the transition of hair follicles into the anagen phase. The anagen ratio was approximately three-fold lower in mice that received testosterone than in normal mice (0.53 vs. 1.55, *p* < 0.0001). In contrast, finasteride or HA–iMSC–EVs led to an increase of anagen ratio (1.29 and 1.19, respectively). No increase in the anagen phase ratio was observed in mice treated with iMSC–EVs (Fig. 5B).

### Regulation in the expression of AR and β-catenin by HA–iMSC–EVs in testosterone-treated AGA mice

**Fig. 6:**
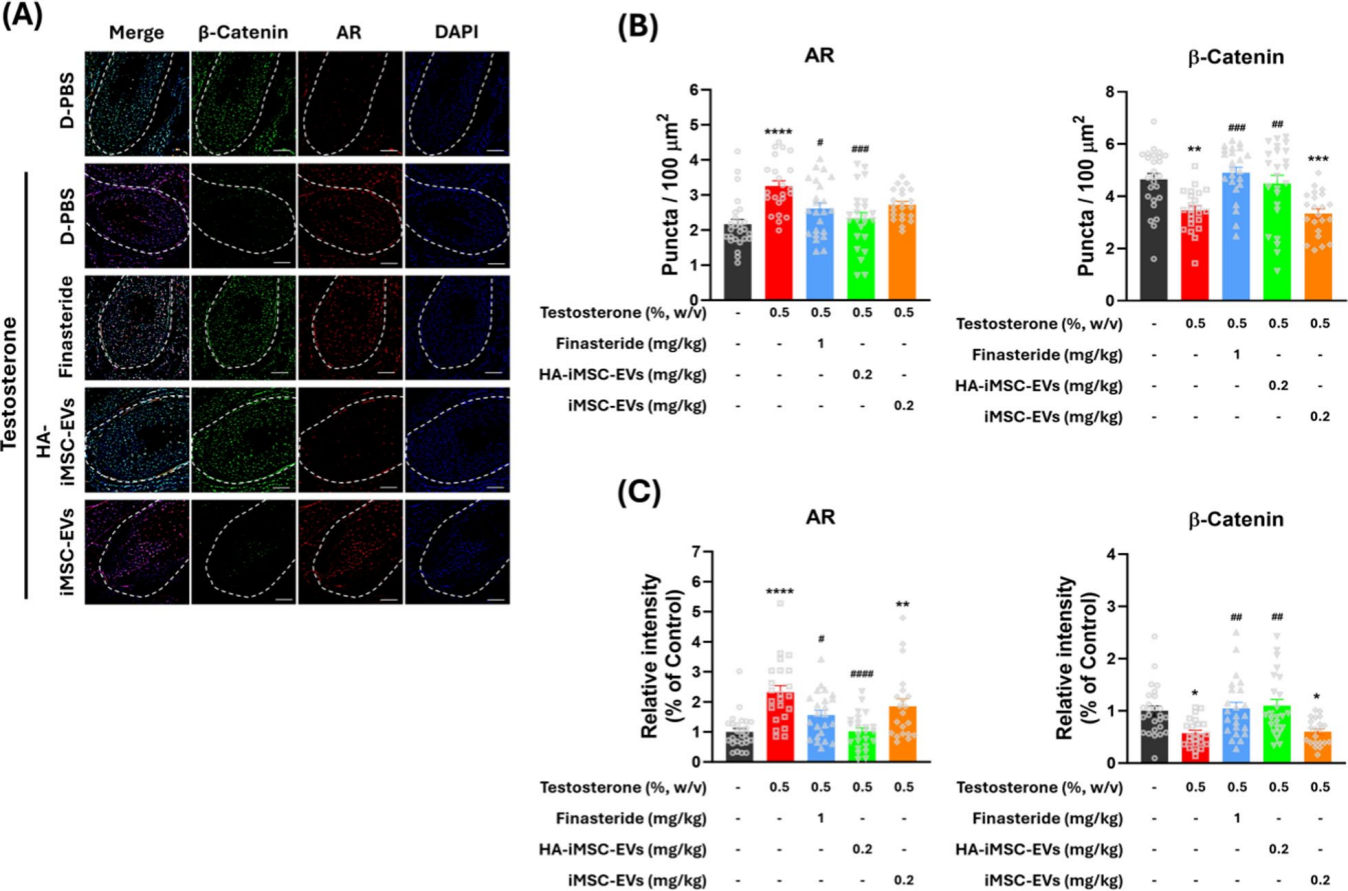
Immunofluorescence detection of androgen receptor (AR) and β-catenin in the hair follicle of AGA mice. **A** Representative images of AR and β-catenin expression in AGA skin tissues. The outline of the hair follicle is indicated by a dotted line. Red and green colors indicate AR and β-catenin, respectively. Scale bars = 25 μm. **B** The AR-positive and β-catenin-positive cells were counted in the form of puncta in the hair follicle area. **C** Quantification of the relative expression of AR and β-catenin in the hair follicle area. **B**, **C** The symbols represent the value of all individual hair follicles counted in five mice. Quantified values from each hair follicle are indicated by gray symbols in **B** and **C**. *p < 0.05, **p < 0.01, ***p < 0.001, ****p < 0.0001 versus vehicle control mice. ^#^*p* < 0.05, ^##^*p* < 0.01, ^###^*p* < 0.001, ^####^*p* < 0.0001 versus testosterone-only group. AGA, androgenetic alopecia

Finally, we conducted immunohistochemical analysis on the expression of AR and β-catenin in the hair follicles of AGA mice (Fig. 6A). In AGA mice, AR-positive puncta and AR intensity was increased, while less β-catenin-positive puncta and β-catenin intensity was detected in AGA mice. In contrast, finasteride and HA–iMSC–EVs reduced the number of AR-positive puncta and AR intensity. Consistently, β-catenin-positive puncta and its intensity was increased by finasteride and HA–iMSC–EVs, while remaining unchanged by iMSC–EVs (Fig. 6B–C). These data indicate that HA–iMSC–EVs have the potential to improve AGA by reducing AR and re-activating β-catenin in AGA skin.

## Discussion

The canonical Wnt/β-catenin signaling is responsible for various physiological processes, such as apoptosis, cell proliferation, differentiation, and tissue homeostasis [42–47]. Wnt/β-catenin signaling can cause AGA [48–51]. Wnt3a-mediated DHT inhibition of proliferating keratinocytes was the first study to emphasize the molecular crosstalk between androgens and Wnt signaling in AGA. Upon AR activation via androgen binding, hair loss is accelerated via a further increase in AR [51, 52], and AR expression was higher in DPCs from AGA males than in DPCs from non-AGA males [53]. Additionally, AR signals activate GSK3β, inhibiting the Wnt pathway by phosphorylating β-catenin and promoting its degradation by proteasomes in DPCs [54, 55]. Thus, it is considered that a balance between the Wnt/β-catenin and AR signaling is important for reducing AGA. Similar to our findings, adipose tissue-derives stem cell (ADSC)-derived exosomes also reduced DHT-induced AGA by inhibiting GSK3β and increasing the expression of key growth factors (VEGF, FGF7, and IGF1); ADSC-derived exosomes restored the proliferation, migration, and hair inducibility of DPCs under DHT-treated condition in vitro and in vivo [19]. However, whether these effects were due to decreased desensitization to androgens (e.g., via reduced AR expression) was not examined.

Increased expression of AR in DPC and prostate cancer cells promotes cellular senescence [52, 56]. In addition, AKT signaling is involved in androgen-AR signaling, promoting DNA damage-mediated early senescence in the DPC of patients with AGA [52]. Proteomic analysis showed that signaling molecules for tissue regrowth (e.g., PI3K/AKT) is enriched in the HA–iMSC–EV proteome signature, and it is possible that multiple molecules (e.g., proteins and microRNAs) may have contributed to the therapeutic outcome. Further studies using mice deficient in these receptors or intermediate signaling molecules are required to better delineate the downstream effects of HA–iMSC–EVs in AGA skin tissue.

EVs from non-MSCs have the potential to treat hair loss. Platelet-rich plasma exosomes improve DPC proliferation and migration of DPCs [57]. Kim et al. [58] also reported that exosomes from bovine colostrum promoted the transition from telogen-to-anagen phase, contributing to hair regrowth to a level comparable to those treated with minoxidil. Additionally, DP spheroid–derived exosomes containing *miR-218-5p* promoted hair regeneration by upregulating β-catenin signaling through a downregulation of the secreted frizzled-related protein 2 (SFRP2), a WNT signaling inhibitor [59]. Rajendran et al. [60] also showed that EVs from fibroblasts harboring Wnt3a promoted migration, proliferation, and elongation of the hair shaft in human hair follicles. Macrophage-derived EVs promote hair growth by inducing the expression of VEGF and keratinocyte growth factor (KGF) [61]. Given the efficacy of EVs produced by various parental cells in AGA, it is important to systematically compare the differences between EVs from parental cells of various origins. Although these studies have shown that EVs are beneficial for the activation of DPCs and regeneration of hair follicles, preparing large amounts of EVs is challenging, and their effects on androgen-induced AGA are unclear. In the present study, we demonstrated that HA stimulation of iMSCs can produce EVs that have enhanced potential for AGA treatment than those from unstimulated iMSCs. Our data showed that HA–iMSC–EVs reduce AR expression and stimulate Wnt signaling via inactivating GSK3β in DPCs. These bimodal functions of HA–iMSC–EVs may have contributed to an enhanced therapeutic effect comparable to that of finasteride in AGA.

Several drugs have been tested for the treatment of AGA; however, their side effects remain a critical problem. Finasteride is the most commonly prescribed FDA-approved drug for the treatment of AGA. However, this drug has been reported to increase the incidence of sexual dysfunction including impotence, decreased libido, and ejaculation disorders in males [62]. In females, oral or systemic finasteride cannot be used due to the hormonal side effects that cause female pattern hair loss (FPHL), a common form of AGA in female. Accordingly, the FDA defines finasteride as a Category X drug that cannot be prescribed to pregnant women [63–65]. Topical finasteride, which has not yet been approved by the FDA, has improved tolerability and can be administered to women; however, its weakness in lowering DHT levels remains [66]. Flutamide, a selective AR antagonist used primarily for prostate cancer, has been tested for the treatment of FPHL in women [67, 68]. Although flutamide was found to be effective in FPHL, it can cause hepatotoxicity and gastrointestinal symptoms at high (750 mg/d) and low (below 350 mg/d) doses [67–70]. Bicalutamide, another selective AR blocker, was also effective in patients with FPHL, with fewer side effects than flutamide in a pilot study [71]. However, an elevation of liver enzyme was observed in 12.5% of patients [68, 71]. Owing to these systemic adverse effects, their use in AGA has been limited. Alternative strategies for AGA have also been tested experimentally. AR-targeting small interfering RNAs markedly reduced AR expression and prevented DHT-mediated changes in C57BL/6 mice [72]. Additionally, AR-targeted self-assembled micelle inhibitory RNA, a novel RNAi with reduced adverse immune responses, was effective in reducing AGA. A study that 48 and 60 patients who were treated with low-dose (0.5 mg/mL, three times/week) and high-dose (5 mg/mL, once a week), respectively, showed meaningful therapeutic outcomes in AGA. However, several side effects including erythema, edema, and local itching were observed in the high-dose group [73].

Although the trophic effect of HA–iMSC–EVs on hair growth has been demonstrated, this study has some limitations. First, the effect of HA–iMSC–EVs on neighboring cell (e.g., immune cells and fibroblasts) production of cytokines and growth factors [74–76] remains to be determined. Second, another valuable aspect of hair growth is capillary formation around the dermis; thus, the effect of HA–iMSC–EVs on the growth or apoptosis of dermal endothelial cells is required [77, 78]. Third, the mechanisms underlying downregulation of AR and how GSK3β is reduced remains to be further determined. Nonetheless, the results of this approach provide key information that will assist in the development of a cell-free method for AGA treatment.

## Conclusion

**Fig. 7:**
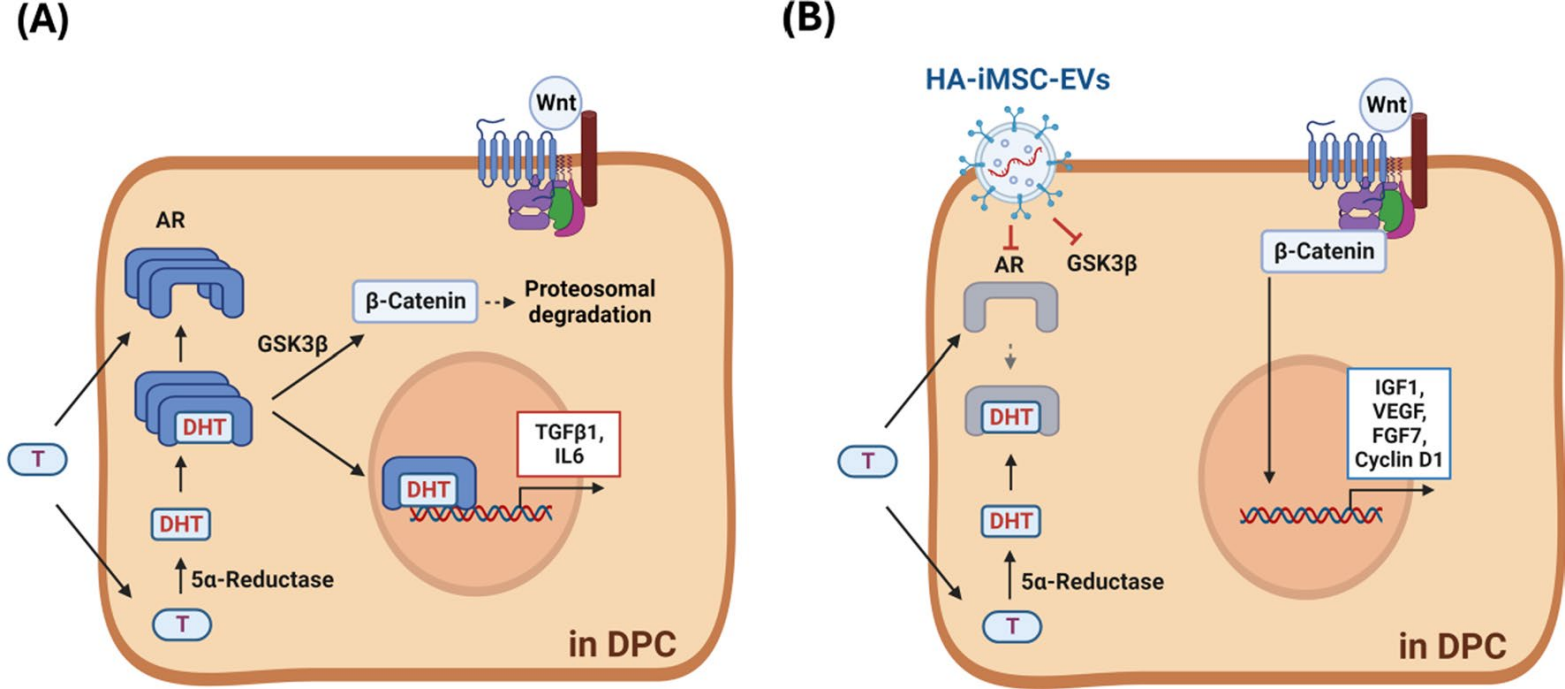
Schematics of action of HA–iMSC–EVs in DPCs. **A** Under testosterone (T)-rich condition, AR expression and DHT production increase, promoting GSK3β-dependent β-catenin degradation and increasing the catagen inducers. **B** HA–iMSC–EVs decrease testosterone-induced AR activation and inhibit GSK3β-dependent β-catenin degradation. Subsequently, the expression of anagenic growth factors is promoted, suppressing and testosterone-induced hair loss. DHT, Dihydrotestosterone; DPC, dermal papilla cell; EV, extracellular vesicle; HA, hyaluronic acid; iMSC, induced pluripotent stem cell-derived mesenchymal stem cell

In this study, we demonstrated that HA treatment of iMSCs produced EVs with an enhanced ability to reduce AGA. HA–iMSC–EVs enhance the viability of DPC undergoing testosterone-induced cell death. HA–iMSC–EVs promoted the mRNA expression of key growth factors, while blocking the transcription of AR, TGFβ1, and IL-6. In AGA mice, the reduction of hair follicular β-catenin protein level in AGA mice were reversed by HA–iMSC–EVs. Mechanistically, HA–iMSC–EVs reduced AR expression and the GSK3β stability, both of which are important players in AGA. Overall, our data suggest that HA–iMSC–EVs promote anagen transition and re-activate hair growth by regulating the expression of growth factors/cytokines, reducing AR, and activating Wnt/β-catenin signaling. To conclude, we demonstrated that HA–iMSC–EVs can reduce AGA progression by suppressing testosterone-induced AR expression and activating Wnt/β-catenin by blocking GSK3β activity (Fig. 7A, B). Thus, our strategy may serve as an alternative treatment option for patients with AGA.

## Supplementary Information

Supplementary Material 1

## Data Availability

Data and materials can be provided to the corresponding author via email upon request.

## Abbreviations

ASC: Adipose-derived stem cells
AGA: Androgenetic alopecia
AR: Androgenic receptor
DPCs: Dermal papilla cells
DKK-1: Dickkopf-1
DHT: Dihydrotestosterone
EGF: Epidermal growth factor
EV: Extracellular vesicle
FPHL: Female pattern hair loss
FGF7: Fibroblast growth factor 7
GSK3β: Glycogen synthase kinase-3β
HFDPC: Hair follicle dermal papilla cells
rhDKK-1: Human recombinant Dickkopf-1
HA-iMSCs: Hyaluronic acid-stimulated iMSCs
iMSCs: Induced pluripotent stem cell-derived MSCs
IGF-1: Insulin like growth factor-1
IL-6: Interleukin-6
MPHL: Male pattern hair loss
MSCs: Mesenchymal stem cells
SC: Subcutaneous
TGFβ1: Transforming growth factor beta1
TSG101: Tumor Susceptibility Gene 101
VEGF: Vascular endothelial growth factor
