# Supplementary material for: Improvement of androgenic alopecia by extracellular vesicles secreted from hyaluronic acid-stimulated induced mesenchymal stem cells
Source: Stem Cell Res Ther. 2024 Sep 11;15:287. doi: 10.1186/s13287-024-03906-x (PMC11389250; doi:10.1186/s13287-024-03906-x)
Supplement: Supplementary file 1 — Supplementary Material 1 [file 13287_2024_3906_MOESM1_ESM.docx]

**Additional information**

**Improvement of androgenic alopecia by extracellular vesicles secreted from hyaluronic acid-stimulated induced mesenchymal stem cells**

Hyun Geun Oh^1^, Minyoung Jung^1^, Seon-Yeong Jeong^1^, Jimin Kim^1^, Sang‑Deok Han^1^, Hongduk Kim^2^, Seulki Lee^1^, Yejin Lee^1^, Haedeun You^1^, Somi Park^1^, Eun A Kim^1^, Tae Min Kim^2,3^*, Soo Kim^1^*

^1^R&D Center, Brexogen Inc., Songpa-gu, Seoul 05855, Republic of Korea

^2^Institutes of Green Bio Science & Technology, Seoul National University, Pyeongchang, Gangwon-do 25354, Republic of Korea

^3^Graduate School of International Agricultural Technology, Seoul National University, Pyeongchang, Gangwon-do 25354, Republic of Korea

*Correspondence to:

Tae Min Kim, D.V.M., Ph.D.

1447 Pyeongchang Daero, Gangwon-do 25254, Republic of Korea

Tel) +82-33-339-5896, Fax) +82-33-339-5689

E-mail: taemin21@snu.ac.kr

Soo Kim Ph.D.

3F, 9, Beobwon-ro 8-gil, Songpa-gu, Seoul, 05855, Republic of Korea

Tel) +82-2-430-0401, Fax) +82-2-430-0402

E-mail: sue.kim@brexogen.com

**Supplemental Methods and Methods**

**Cell viability assay**

Cell Counting Kit-8 (Enzo) was used for the cell viability assay. To measure HFDPC viability against testosterone, 1.5 × 10^3^ HFDPCs were seeded into a 96-well plate and cultured in complete medium. After a 24 h incubation, the medium was removed from each well. Cells were washed twice in DPBS and treat with 50 μM testosterone in a serum-free DMEM. The cells were then added with HA-iMSC-EVs or iMSC-EVs (10, 25, and 50 μg/mL). After 48 h, the CCK-8 solution was added, and the optical density was measured at 450 nm.

**Supplementary Tables**

Table S1. List of antibodies used for western blotting.

| **Antigen** | **Host** | **Type** | **Dilution** | **Supplier**  **(Catalog no.)** |
| --- | --- | --- | --- | --- |
| CD63 | Rabbit | Polyclonal | 1:200 | Abcam  (ab216130) |
| CD81 | Mouse | Monoclonal | 1:250 | Invitrogen  (10630D) |
| TGS101 | Rabbit | Monoclonal | 1:1000 | Invitrogen  (MA5-32463) |
| GAPDH | Rabbit | Polyclonal | 1:5000 | Abcam  (ab9485) |
| AR | Mouse | Monoclonal | 1:500 | Santa Cruz Biotechnology  (sc-7305) |
| β-catenin | Rabbit | Polyclonal | 1:1000 | Cell Signaling Technology  (#9562) |
| GSK3β | Rabbit | Monoclonal | 1:2000 | Cell Signaling Technology  (#12456) |
| phospho-GSK3β | Rabbit | Monoclonal | 1:1000 | Cell Signaling Technology  (#5558) |
| Cyclin D1 | Rabbit | Monoclonal | 1:2000 | Cell Signaling Technology  (#55506) |
| Lamin A/C | Rabbit | Polyclonal | 1:2000 | Cell Signaling Technology  (#2032) |
| Rabbit-IgG | Goat | Polyclonal,  HRP-conjugated | 1:2000 | Abcam  (ab6721) |
| Mouse-IgG | Goat | Polyclonal,  HRP-conjugated | 1:2000 | Abcam  (ab205719) |

Table S2. Sequence of primers used for quantitative RT-PCR.

| **Target gene** | **Sequences** |
| --- | --- |
| Human *AR* | F: CCT GGC TTC CGC AAC TTA CAC  R: GGA CTT GTG CAT GCG GTA CTC A |
| Human *TGFβ1* | F: CCC AGC ATC TGC AAA GCT C  R: GTC AAT GTA CAG CTG CCG CA |
| Human *IL-6* | F: ACA GCC ACT CAC CTC TTC AG  R: CCA TCT TTT TCA GCC ATC TTT |
| Human *IGF1* | F: CTT CAG TTC GTG TGT GGA GAC AG  R: CGC CCT CCG ACT GCT G |
| Human *FGF7* | F: TCC TGC CAA CTT TGC TCT ACA  R: CAG GGC TGG AAC AGT TCA CAT |
| Human *VEGF* | F: CTA CCT CCA CCA TGC CAA GT  R: GCA GTA GCT GCG CTG ATA GA |
| Human *GAPDH* | F: GTC GG AGTC AACGGA TTT GG  R: AGT TGA GGT CAA TGA AGG GGT C |

Table S3. List of antibodies used for immunohistochemistry.

| **Antigen** | **Host** | **Type** | **Dilution** | **Supplier**  **(Catalog no.)** |
| --- | --- | --- | --- | --- |
| AR | Mouse | Monoclonal | 1:100 | Santa Cruz Biotechnology  (sc-7305) |
| β-catenin | Rabbit | Polyclonal | 1:300 | Cell Signaling Technology  (#9562) |
| N/A | Goat | Anti-rabbit IgG,  Polyclonal,  Alexa fluor 488-conjugated | 1:500 | Abcam  (150077) |
| N/A | Goat | Anti-mouse IgG,  Polyclonal,  Cyanin5-conjugated | 1:500 | Invitrogen  (A10524) |

N/A: non-applicable

Table S4. List of differentially expressed proteins in HA-iMSC-EVs.

| **Pathways** | **Gene symbol** | **Protein accession No.** | **Protein name** | ***P-*value** | **Log_2_FC** | **FDR q-value** |
| --- | --- | --- | --- | --- | --- | --- |
| **Pathways** | **Gene symbol** | **Protein accession No.** | **Protein name** | ***P-*value** | **Log_2_FC** | **FDR q-value** |
| **ECM-receptor interaction** | COL4A1 | P02462 | Collagen alpha-1(IV) chain | 0.0007 | 0.6323 | q = 0.004 q = 0.002 |
|  | COL4A2 | P08572 | Collagen alpha-2(IV) chain | 0.0026 | 0.433 |  |
|  | COL4A5 | P29400 | Collagen alpha-5(IV) chain | 0.0005 | 0 |  |
|  | COL6A3 | P12111 | Collagen alpha-3(VI) chain | 0.006 | -0.2141 |  |
|  | THBS1 | P07996 | Thrombospondin-1 | 0.0008 | 0.4751 |  |
|  | ITGA5 | P08648 | Integrin alpha-5 | 0.0021 | -0.6323 |  |
|  | LAMA1 | P25391 | Laminin subunit alpha-1 | 0.0026 | -0.4542 |  |
|  | LAMA2 | P24043 | Laminin subunit alpha-2 | 0.0003 | -0.8875 |  |
|  | LAMA4 | Q16363 | Laminin subunit alpha-4 | < 0.0001 | -0.9928 |  |
|  | LAMB1 | P07942 | Laminin subunit beta-1 | 0.0001 | -0.5558 |  |
|  | LAMC1 | P11047 | Laminin subunit gamma-1 | < 0.0001 | -0.5656 |  |
|  | LAMC2 | Q13753 | Laminin subunit gamma-2 | 0.0036 | 0 |  |
|  | ITGB1 | P05556 | Integrin beta-1 | 0.0013 | -1.275 |  |
|  | CD44 | P16070 | CD44 antigen | 0.001 | -0.696 |  |
| **PI3K/AKT signaling** | COL4A1 | P02462 | Collagen alpha-1(IV) chain | 0.0007 | 0.6323 | q = 0.038 q = 0.237 |
|  | COL4A2 | P08572 | Collagen alpha-2(IV) chain | 0.0026 | 0.433 |  |
|  | COL4A5 | P29400 | Collagen alpha-5(IV) chain | 0.0005 | 0 |  |
|  | THBS1 | P07996 | Thrombospondin-1 | 0.0008 | 0.4751 |  |
|  | ITGA5 | P08648 | Integrin alpha-5 | 0.0021 | -0.6323 |  |
|  | ITGB1 | P05556 | Integrin beta-1 | 0.0013 | -1.275 |  |
|  | GNG12 | Q9UBI6 | Guanine nucleotide-binding protein G(I)/G(S)/G(O) subunit gamma-12 | 0.0006 | -1.4005 |  |
|  | LAMA1 | P25391 | Laminin subunit alpha-1 | 0.0026 | -0.4542 |  |
|  | LAMA2 | P24043 | Laminin subunit alpha-2 | 0.0003 | -0.8875 |  |
|  | LAMA4 | Q16363 | Laminin subunit alpha-4 | < 0.0001 | -0.9928 |  |
|  | LAMB1 | P07942 | Laminin subunit beta-1 | 0.0001 | -0.5558 |  |
|  | LAMC1 | P11047 | Laminin subunit gamma-1 | < 0.0001 | -0.5656 |  |
|  | LAMC2 | Q13753 | Laminin subunit gamma-2 | 0.0036 | 0 |  |
|  | CSF1 | P09603 | Macrophage colony-stimulating factor 1 | 0.017 | -0.585 |  |
|  | COL6A3 | P12111 | Collagen alpha-3(VI) chain | 0.006 | -0.2141 |  |
|  | RAC1 | P63000 | Ras-related C3 botulinum toxin substrate 1 | 0.0006 | -1.2079 |  |
|  | YWHAZ | P63104 | 14-3-3 protein zeta/delta | 0.0003 | -0.3674 |  |
|  | RPS6 | P62753 | 40S ribosomal protein S6 | 0.0016 | -0.6781 |  |
| **Proteosome** | PSMA1 | P25786 | Proteasome subunit alpha type-1 | 0.0002 | -0.585 | q  = 4.86E-26 |
|  | PSMA2 | P25787 | Proteasome subunit alpha type-2 | 0.0016 | -0.5656 |  |
|  | PSMA4 | P25789 | Proteasome subunit alpha type-4 | 0.001 | -0.4751 |  |
|  | PSMA5 | P28066 | Proteasome subunit alpha type-5 | 0.0013 | -0.7824 |  |
|  | PSMA6 | P60900 | Proteasome subunit alpha type-6 | 0.0004 | -0.546 |  |
|  | PSMA7 | O14818 | Proteasome subunit alpha type-7 | 0.0012 | -0.5059 |  |
|  | PSMB1 | P20618 | Proteasome subunit beta type-1 | 0.0009 | -0.3448 |  |
|  | PSMB2 | P49721 | Proteasome subunit beta type-2 | 0.0011 | -0.4647 |  |
|  | PSMB4 | P28070 | Proteasome subunit beta type-4 | 0.0019 | -0.669 |  |
|  | PSMB6 | P28072 | Proteasome subunit beta type-6 | 0.0006 | -0.8319 |  |
|  | PSMB7 | Q99436 | Proteasome subunit beta type-7 | < 0.0001 | -0.3674 |  |
|  | PSMB8 | P28062 | Proteasome subunit beta type-8 | 0.0043 | -0.3103 |  |
|  | PSMC2 | P35998 | 26S proteasome regulatory subunit 7 | 0.0008 | -0.7137 |  |
|  | PSMC3 | P17980 | 26S proteasome regulatory subunit 6A | 0.0015 | -0.4854 |  |
|  | PSMC5 | P62195 | 26S proteasome regulatory subunit 8 | 0.0023 | -0.6871 |  |
|  | PSMC6 | P62333 | 26S proteasome regulatory subunit 10B | 0.0032 | -0.9486 |  |
|  | PSMD1 | O00231 | 26S proteasome non-ATPase regulatory subunit 11 | 0.0007 | -0.848 |  |
|  | PSMD2 | Q13200 | 26S proteasome non-ATPase regulatory subunit 2 | 0.0011 | -0.3561 |  |
|  | PSMD6 | Q15008 | 26S proteasome non-ATPase regulatory subunit 6 | 0.0006 | -0.9486 |  |
|  | PSMD7 | P51665 | 26S proteasome non-ATPase regulatory subunit 7 | 0.0013 | -0.6871 |  |
|  | PDMD8 | P48556 | 26S proteasome non-ATPase regulatory subunit 8 | < 0.0001 | -1.0566 |  |
|  | PDMD11 | O00231 | 26S proteasome non-ATPase regulatory subunit 11 | 0.0007 | -0.4222 |  |
|  | PSMD12 | O00232 | 26S proteasome non-ATPase regulatory subunit 12 | < 0.0001 | -0.6781 |  |
|  | PSMD13 | Q9UNM6 | 26S proteasome non-ATPase regulatory subunit 13 | 0.0002 | -0.84 |  |
|  | PSMD14 | O00487 | 26S proteasome non-ATPase regulatory subunit 14 | 0.0005 | -0.9928 |  |


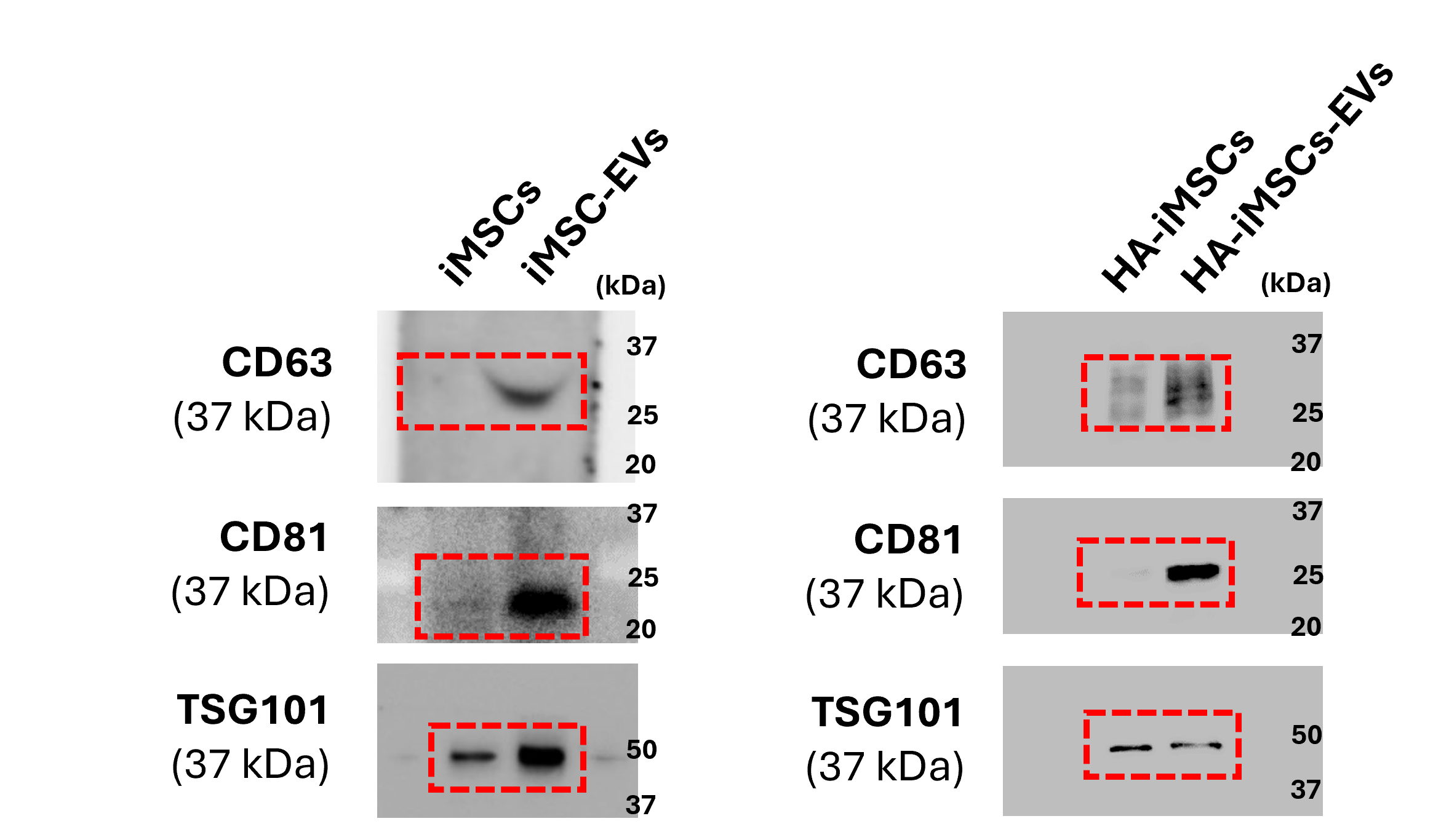


**Figure S1.** Uncropped images of western blotting for Figure 1 (B). The red boxes indicate the cropped images of western blotting shown in Figure 1 (B). Membranes were cut to enable blotting for multiple antibodies.


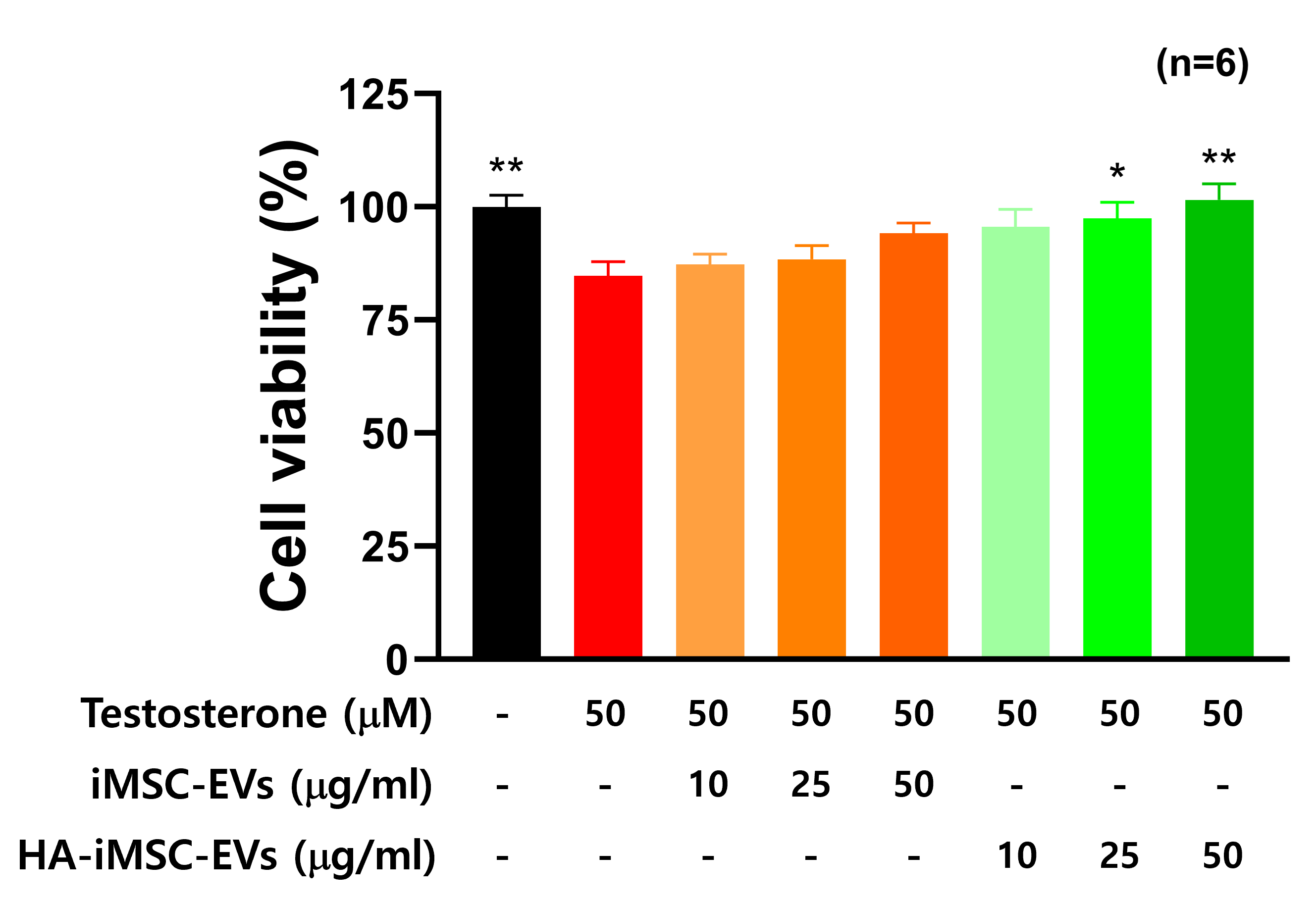


**Figure S2.** HA-iMSC-EVs recovered cell viability in testosterone-induced androgenic alopecia in vitro model. (A) The viability of HDFPC was measured after being cultured with 10, 25 and 50 μg/ml iMSC-EVs or HA-iMSC-EVs. *p<0.05, **p<0.01 vs. testosterone-treated group. EV, extracellular vesicle; HA, hyaluronic acid; HFDPC, hair follicle dermal papilla cell; iMSC, induced pluripotent stem cell-derived mesenchymal stem cell


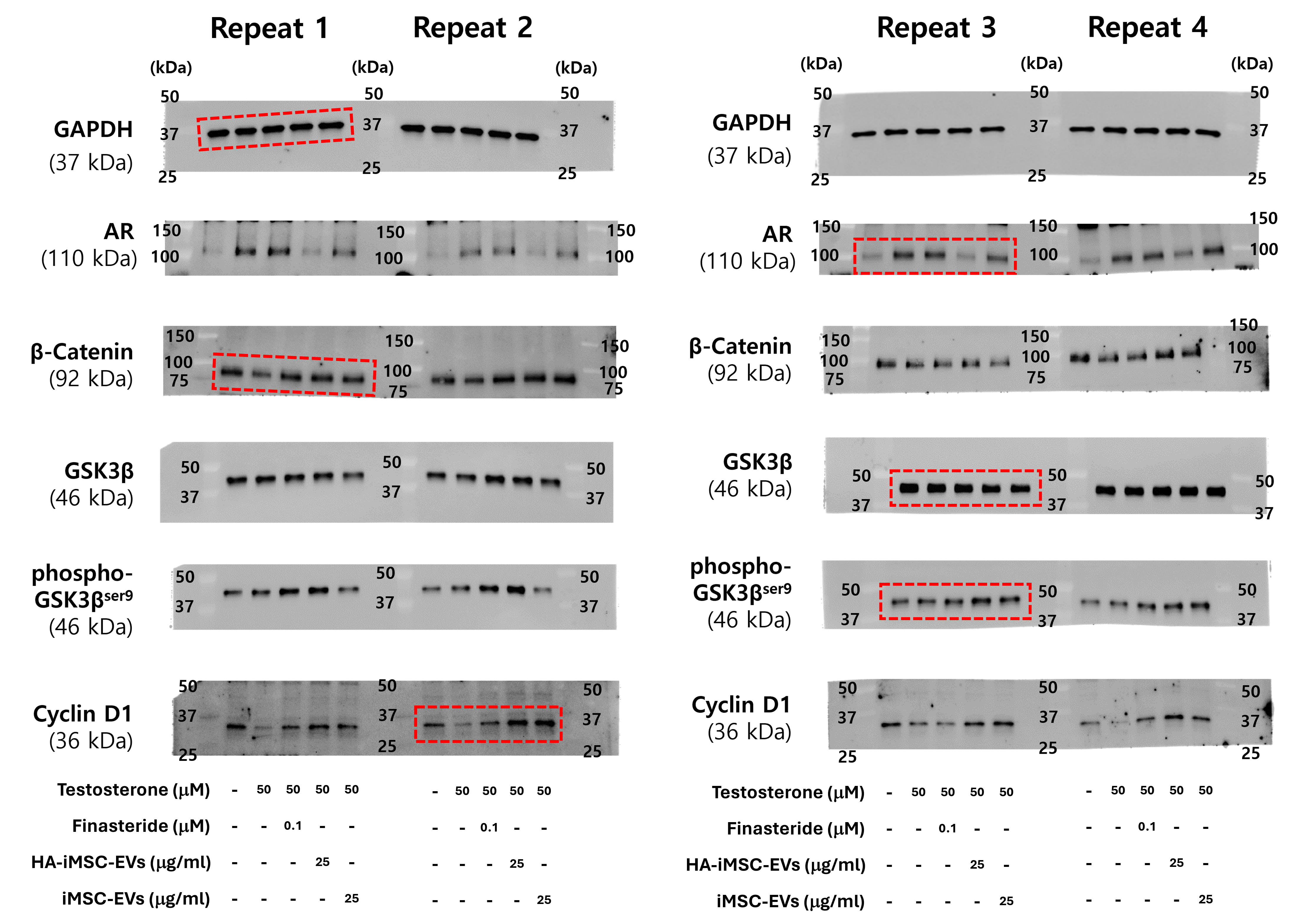


**Figure S3.** Uncropped images of western blotting for Figure 3 (A-E). The red boxes indicate the cropped images of western blotting shown in Figure 3 (A). Membranes were cut to enable blotting for multiple antibodies.


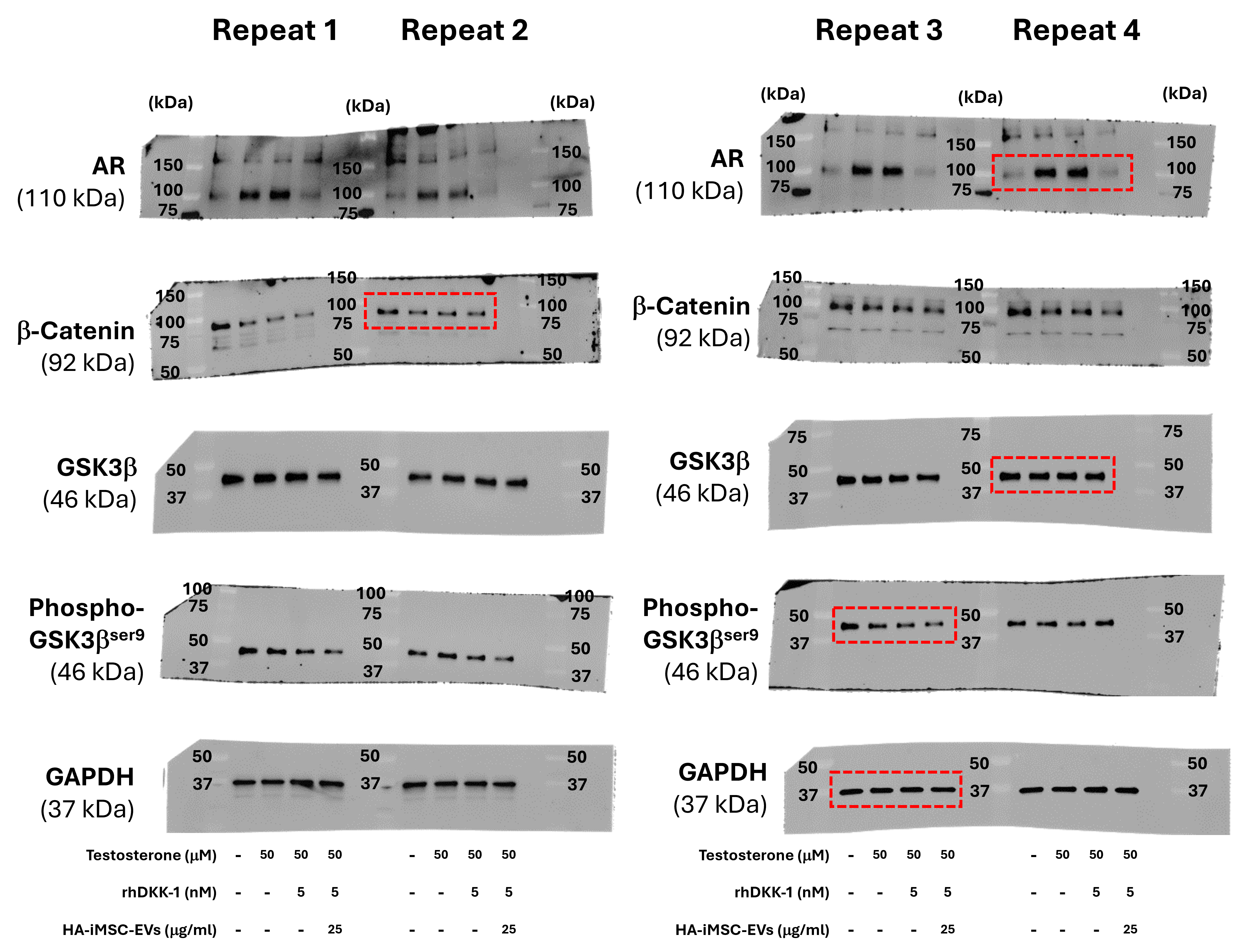


**Figure S4.** Uncropped images of western blotting for Figure 3 (F-I). The red boxes indicate the cropped images of western blotting shown in Figure 3 (F). Membranes were cut to enable blotting for multiple antibodies.


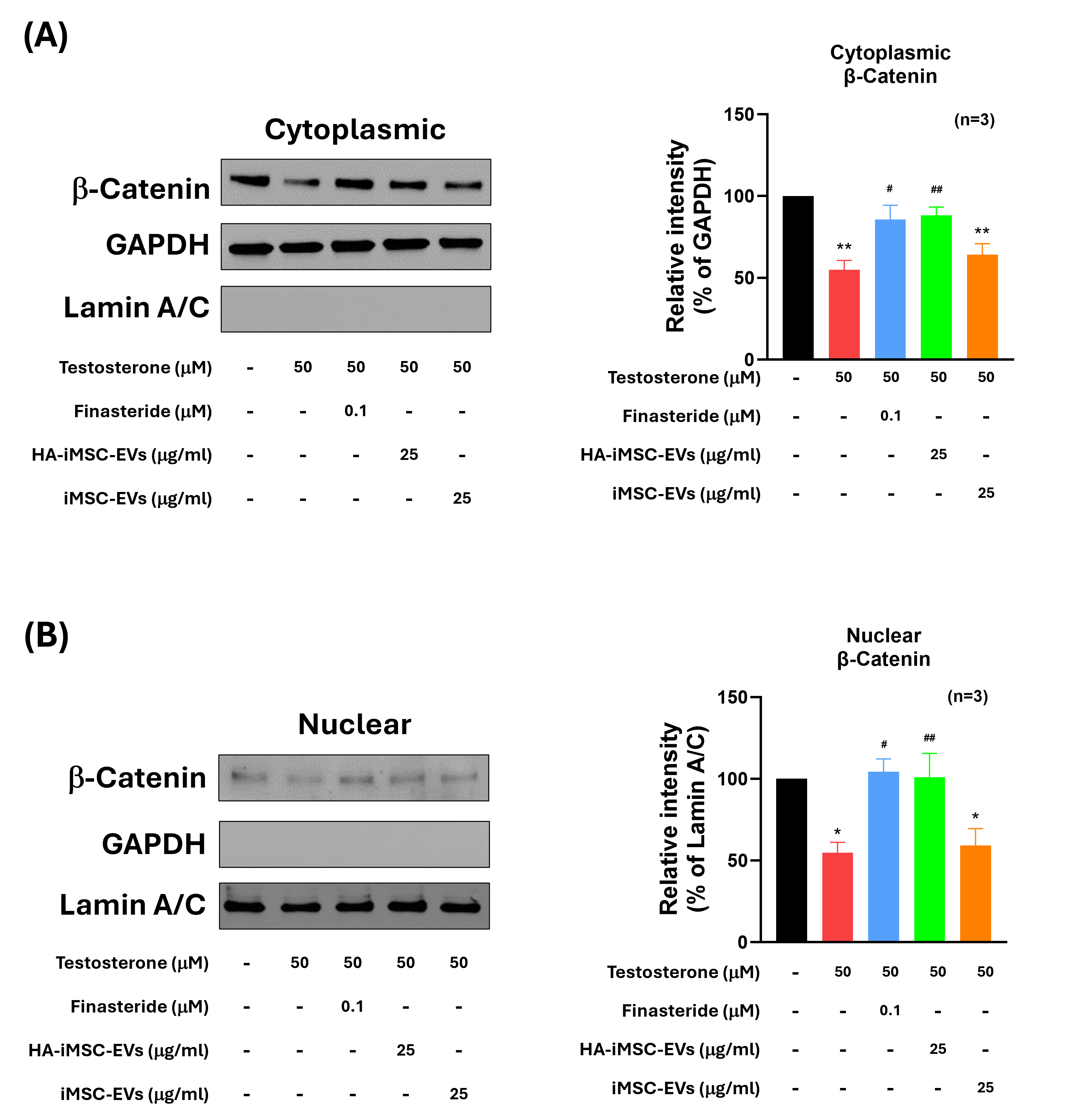


**Figure S5.** Translocation of testosterone-reduced β-catenin to the nucleus was restored by finasteride or HA-iMSC-EVs. The cytoplasmic β-catenin expression (A) and the nuclear β-catenin expression (B) were normalized to the loading control, GAPDH and Lamin A/C, respectively, and were replicated from three independent experiments. Uncropped western blot images are shown in Additional file 1: Figure S6. *p<0.05, **p<0.01 vs. control group. ^#^p<0.05, ^##^p<0.01 vs. testosterone-only group. EV, extracellular vesicle; HA, hyaluronic acid; HFDPC, hair follicle dermal papilla cell; iMSC, induced pluripotent stem cell-derived mesenchymal stem cell


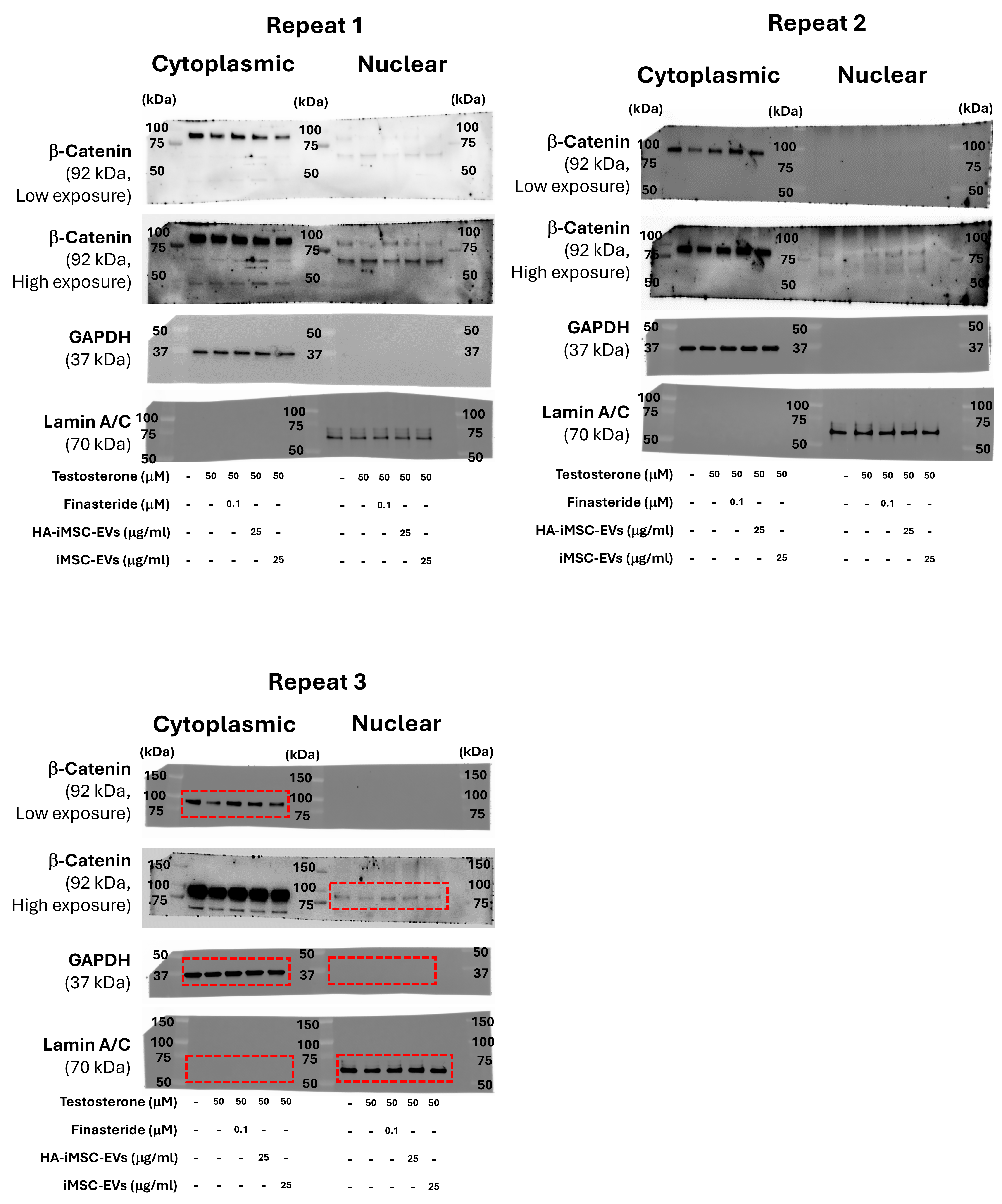


**Figure S6.** Uncropped images of western blotting for Figure S5 (A, B). The red boxes indicate the cropped images of western blotting shown in Figure S5 (A, B). Membranes were cut to enable blotting for multiple antibodies.
